# Long-Term Evolution of Myocardial Strain and Coronary Artery Z-Scores in Multisystem Inflammatory Syndrome in Children *Versus* Kawasaki Disease

**DOI:** 10.3390/children13060813

**Published:** 2026-06-12

**Authors:** Naqiya Arsiwala, Anoushka Krishnakumar, Yutika Chirlikar, Donato Rigante

**Affiliations:** 1 Department of Life Sciences and Public Health, Fondazione Policlinico Universitario A. Gemelli IRCCS, Largo A. Gemelli 8, I-00168 Rome, Italy; naqiyashabbir.arsiwala01@icatt.it (N.A.); anoushka.krishnakumar01@icatt.it (A.K.); yutikasatisj.chirlikar01@icatt.it (Y.C.); 2 Periodic Fevers Research Center, Università Cattolica Sacro Cuore, I-00168 Rome, Italy

**Keywords:** multisystem inflammatory syndrome in children (MIS-C), Kawasaki disease, global longitudinal strain, subclinical myocardial dysfunction, coronary artery Z-score, personalized medicine

## Abstract

**Highlights:**

**What are the main findings?**

- In MIS-C acute diffuse myocardial dysfunction with severely impaired global longitudinal, circumferential, and atrial strain predicts adverse cardiovascular outcomes independently of ejection fraction, with right ventricular recovery lagging left ventricular recovery at six months.
- Kawasaki disease imposes persistent subclinical myocardial deformation abnormalities detectable only by speckle-tracking echocardiography, correlating with fibrosis biomarkers, despite angiographic and functional normalization.

**What are the implications of the main findings?**

- The divergent cardiac trajectories of MIS-C and Kawasaki disease demand condition-specific surveillance frameworks: strain-centred follow-up for MIS-C and structured coronary Z-score monitoring for Kawasaki disease.
- Ejection fraction systematically underestimates residual myocardial injury in both conditions, making strain imaging not merely adjunctive but essential to risk stratification in pediatric post-inflammatory heart disease.

**Abstract:**

Multisystem inflammatory syndrome in children (MIS-C) and Kawasaki disease (KD) are pediatric inflammatory conditions which share overlapping clinical features, yet their long-term cardiovascular trajectories remain incompletely characterized. Understanding differences in myocardial strain evolution and coronary artery Z-score progression is essential for optimizing surveillance strategies and risk stratification. Aims of this review were to comprehensively compare the long-term evolution of myocardial strain parameters and coronary artery Z-scores in children with MIS-C *versus* KD through mid- and long-term follow-up assessment studies, and to identify clinical implications for monitoring and management. A comprehensive literature search was conducted in PubMed to identify studies evaluating myocardial strain and coronary artery Z-scores in MIS-C and KD. Publications from January 2020 to February 2026 were considered for MIS-C, with earlier key studies on KD included to contextualize established cardiac outcomes. Observational studies and cohort reports describing echocardiographic findings and follow-up data were reviewed. Available evidence indicates that MIS-C commonly presents with acute myocardial dysfunction, frequently characterized by reduced global longitudinal strain despite preserved or mildly reduced ejection fraction; in most cases, myocardial strain abnormalities substantially improve within weeks to a few months following treatment. In contrast, myocardial strain impairment in KD, which typically presents at less than 5 years of age, is less pronounced; coronary artery involvement shows an opposite trend, as KD is more frequently associated with coronary dilations and aneurysm formation, reflected by persistent elevations in coronary artery Z-scores, whereas coronary abnormalities in MIS-C are milder and often transient. Recovery patterns therefore differ, with MIS-C demonstrating rapid myocardial functional recovery, and KD carrying a greater risk of long-term coronary artery sequelae. MIS-C and KD exhibit distinct cardiovascular phenotypes: MIS-C is primarily characterized by reversible myocardial dysfunction, whereas KD remains a condition most strongly associated with a risk of persistent coronary artery abnormalities. Deciphering these differences may help guide disease-specific cardiac monitoring and long-term follow-up strategies in affected children.

## 1. Introduction

The landscape of pediatric inflammatory diseases has been substantially reshaped by the emergence of multisystem inflammatory syndrome in children (MIS-C), a hyperinflammatory condition temporally associated with severe acute respiratory syndrome coronavirus 2 (SARS-CoV-2) infection [1]. However, MIS-C shares several clinical hallmarks with Kawasaki disease (KD), a well-defined vasculitis which is also the leading cause of acquired heart disease for children living in developed countries, due to its significant risk of cardiovascular sequelae [2,3]. Despite these similarities, MIS-C and KD differ in the nature, severity, and long-term trajectory of their cardiac manifestations, with important implications for post-acute surveillance and management. KD has historically been defined by its predilection for the coronary arteries, with a well-documented remarkable risk of coronary artery aneurysm (CAA) formation if left untreated [4]. MIS-C, by contrast, is more frequently characterized by acute myocardial injury, often presenting with features of cardiogenic shock with hemodynamic instability and markedly elevated cardiac biomarkers, while coronary involvement, when present, tends to be milder and often transient [2,5]. These differing phenotypes suggest that the dominant long-term cardiovascular risk in KD lies in the coronary vasculature, whereas in MIS-C it may reside in the residual myocardial dysfunction. Traditional echocardiographic parameters, such as left ventricular ejection fraction (LVEF), often show rapid recovery in MIS-C survivors, though it may lack sensitivity to detect subclinical myocardial dysfunction in inflammatory conditions [6]. Conversely, the global longitudinal strain (GLS), a simple parameter that expresses longitudinal shortening as a percentage via two-dimensional speckle-tracking echocardiography, has emerged as an important tool for assessing intrinsic myocardial deformation in cardiomyopathies, ischemic heart disease or valvular diseases, and potentially in MIS-C [7]. KD, in contrast, is characterized by myocardial strain changes that are often regional in distribution, predominantly affecting the basal and apical segments [8]. Similarly, coronary artery Z-scores—which standardize coronary dimensions for body surface area—provide a quantitative framework for tracking the evolution of coronary involvement in KD over time, and their long-term trajectories differ if compared to MIS-C. While KD is associated with a higher risk of persistent and severe CAAs, long-term echocardiographic follow-up of MIS-C cohorts demonstrates that coronary artery Z-scores often remain normal or normalize by one year, supporting a limited long-term risk of coronary sequelae for these patients [9]. This review synthesizes current evidence on the longitudinal trajectories of myocardial strain and coronary artery Z-scores in MIS-C *versus* KD, with the aim of detailing evidence-based condition-specific approaches for long-term cardiovascular surveillance in both conditions.

## 2. Materials and Methods

**Figure 1:**
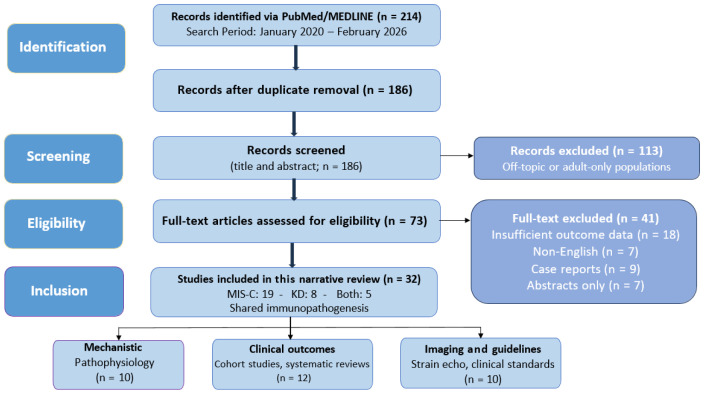
Flow diagram detailing the whole process used for the preparation of this narrative review.

This narrative review has been conducted in accordance with standard methodological principles for unsystematic literature synthesis. No prospective registration was undertaken, consistently with our aims. The literature search was performed using PubMed/MEDLINE, with searches conducted from 2020 to early 2026 to capture eligible published or ahead-of-print publications. Search terms were developed across two primary thematic domains: (1) immunopathogenesis and cardiovascular complications of MIS-C and KD, and (2) myocardial strain imaging in pediatric inflammatory conditions. Core search strings combined the following terms using Boolean operators (AND, OR): “multisystem inflammatory syndrome in children”, “Kawasaki disease”, “coronary artery aneurysm”, “myocardial strain”, “speckle tracking echocardiography”, “global longitudinal strain”, “pediatric myocarditis”, “cardiac outcomes”, “coronary Z-score”, and “post-coronavirus disease (COVID)-19 pediatric patients”. Studies were screened based on both title and abstract, followed by full-text review, where appropriate. Given the narrative design, no formal quality appraisal tool was applied; however, a strict methodological rigour was considered during source selection. A total of 32 references were included in the final synthesis, encompassing prospective cohort studies, multicentre observational studies, mechanistic and single-cell transcriptomic analyses, systematic reviews, echocardiographic outcome studies, and major clinical practice guidelines. Included studies met the following criteria: (i) pediatric populations diagnosed with MIS-C or KD (age <18 years); (ii) assessment of myocardial function using strain imaging and/or evaluation of coronary artery dimensions using Z-scores; (iii) longitudinal or follow-up data on cardiovascular outcomes; (iv) relevant systematic reviews, guidelines, or mechanistic studies providing context for cardiovascular pathophysiology. Exclusion criteria were related to: (i) non-English publications; (ii) studies focusing exclusively on adult populations; (iii) studies lacking cardiac imaging details or without relevance for myocardial and coronary outcomes. All relevant data were extracted qualitatively, including study design, patient population, imaging modalities, and key findings related to myocardial deformation and coronary artery involvement (Figure 1).

## 3. Shared Post-Infectious Phenotype, but Distinct Cardiac Trajectories

Cardiac involvement is a defining feature of both MIS-C and KD, yet with strikingly different predilection and characteristics. In KD, coronary artery abnormalities develop in approximately 20–25% of untreated patients, and timely intravenous immunoglobulin (IVIG) administration reduces this risk to approximately 5% [10]. MIS-C more frequently compromises myocardial function, with ventricular dysfunction reported in a substantial proportion of presentations [2], while coronary dilations may appear up to 30% of cases and tend to be transient and self-resolving [9]. CAA prevalence in MIS-C varies across cohorts, ranging from approximately 16% in large registry-based studies [11] to up to 30% in some prospective multicenter series [9], likely reflecting differences in diagnostic thresholds and population characteristics. This divergence in primary cardiac target is coronary-predominant in KD *versus* myocardium-predominant in MIS-C, which may create a diagnostic paradox: two conditions sharing substantial clinical overlap to complicate acute differentiation yet following fundamentally different structural trajectories [9,11]. Both MIS-C and KD manifest as post-infectious hyper-inflammatory conditions targeting the pediatric cardiovascular system, yet accumulating evidence suggests fundamentally different immunologic mechanisms driving their contrasting long-term cardiovascular outcomes. It should be acknowledged that this comparative framework reflects predominant rather than exclusive phenotypic expression: in fact, both conditions involve overlapping inflammatory mechanisms, including degrees of myocardial inflammation in KD and transient vascular injury in MIS-C, and the myocardial-*versus*-coronary distinction is intended as a clinically useful simplification rather than a rigid biological boundary.

### 3.1. Temporal and Triggering Mechanisms in MIS-C and KD

The timing of MIS-C onset is distinct from an acute viral illness, as symptoms emerge 2–8 weeks following SARS-CoV-2 virus exposure, during a period when antibody responses peak and viral replication has ceased [2,12]. Current understanding attributes this syndrome to a dysregulated immune activation in susceptible hosts, potentially through multiple pathways including molecular mimicry or superantigen-like activation of T-cell populations [2,12]. KD etiology remains enigmatic after five decades of research: despite extensive investigation, no unique causative pathogen has been identified. Current evidence supports an abnormal immune response to one or more environmental as well as infectious triggers in genetically predisposed children, though the precise initiating event is yet unravelled [4,10]. This etiological uncertainty is itself clinically significant: unlike MIS-C, where a defined preceding infection informs immune profiling and surveillance, KD offers no equivalent anchor for predicting which patients will develop severe disease expression or persistent coronary artery involvement.

### 3.2. Vascular Pathology: Divergent Mechanisms of Arterial Injuries in MIS-C and KD

The coronary arteriopathy of KD progresses through three consecutive phases: (a) initial synchronized necrotizing inflammation destroying arterial architecture during the second week after onset and enabling aneurysm formation; (b) subsequent asynchronous chronic inflammation with lymphocytes, plasma cells, and eosinophils persisting months to years; (c) luminal myofibroblastic proliferation (LMP) with potential risk of subsequent stenosis [10]. Histologic examination of KD acute phase reveals neutrophil-driven transmural arterial destruction without granulomatous features, transitioning to intimal thickening and lymphocyte-predominant inflammation in later phases [10,13]. LMP constitutes an ongoing proliferative response involving myofibroblasts, derived from medial smooth muscle cells and their extracellular matrix, explaining why aneurysms appearing “normalized” on imaging may retain substantial pathologic burden and a clinically relevant stenosis risk [13]. Among CAAs, giant aneurysms (≥8 mm diameter or with a Z-score ≥10) undergo remodelling rather than a true resolution, rarely rupturing but frequently developing occlusive thrombi [10]. Mechanisms of vascular injury in MIS-C, while less completely characterized, involve SARS-CoV-2–specific immune complex deposition on the vascular endothelium, triggering downstream inflammation via Fc-γ receptors and complement pathway activation [14]. This immune complex-driven process is also shown by the detection of circulating SARS-CoV-2 immune complexes in patients with MIS-C: the approximately 80% regression rate of coronary abnormalities in MIS-C implies predominantly inflammatory and functional endothelial injury rather than the structural remodelling that defines KD coronary arteriopathy.

### 3.3. Immune Cell Dysregulation and Cytokine Profiles in MIS-C and KD

Single-cell transcriptomic studies identify both convergent and divergent immune signatures. At the level of innate immunity, both MIS-C and KD share expansion of CD177-expressing neutrophils displaying hyperactivated phenotypes, with remarkably similar gene expression patterns which indicate enhanced degranulation capacity and implicate neutrophil-mediated vascular injury as a common upstream mechanism [15]. In contrast to IgA vasculitis, a distinct small-vessel vasculitis mediated by IgA immune complex deposition, in KD there is no established or deciphered pathogenetic mechanism; its vascular injury is driven by innate immune activation and neutrophil-predominant transmural arteritis of medium-sized vessels, a pathological distinction that has direct implications for treatment and long-term monitoring strategies [10]. Critical distinctions emerge for adaptive immunity. MIS-C exhibits pronounced T-cell activation and expansion, particularly of CD8+ populations, despite presenting with lymphopenia, a pattern distinguishable from severe adult COVID-19 [14]. Autoantibody profiling in MIS-C demonstrates reactivity against immune signalling molecules and cardiovascular structural proteins, while T-cell receptor analysis suggests superantigen driven polyclonal activation as a plausible mechanistic driver [12,15,16]. Cytokine analysis reveals overlapping yet distinct inflammatory signatures. In fact, both conditions demonstrate elevation of interferon (IFN) pathway markers as IFN-γ, interleukin (IL)-18, IFN-induced protein 10 (IP-10, also named CXCL10), and monocyte activation indicators as monocyte chemoattractant protein-1 (MCP-1), IL-1α and IL-1 receptor antagonist (IL-1RA) [17]. Computational modelling identifies IL-15/IL-15 receptor antagonist (IL-15RA) signalling as a convergent pathway, suggesting a shared upstream immunopathogenesis despite divergent clinical phenotypes [17]. A distinct MIS-C subgroup (designated MIS-C plus) demonstrates markedly elevated IFN-γ, IL-18, granulocyte-macrophage colony-stimulating factor (GM-CSF), chemokine C-C motif ligand 5 (CCL5, also named RANTES), IP-10, IL-1α, stromal cell-derived factor-1 (SDF-1, also named CXCL12), with labwork features consistent with incipient macrophage activation syndrome [18]. This IFN-γ predominant profile defines a higher-severity MIS-C subphenotype and carries implications for understanding the heterogeneity of cardiac outcomes within MIS-C itself, a heterogeneity that complicates direct comparison with KD and must be accounted for when interpreting long-term strain and Z-score trajectories [17,18].

### 3.4. Mechanisms of Myocardial Injury in MIS-C and KD

**Figure 2:**
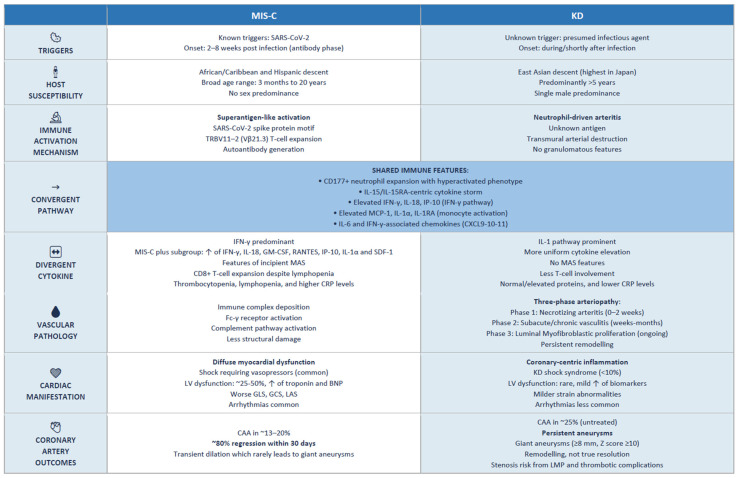
Comparative pathophysiological pathways in multisystem inflammatory syndrome in children (MIS-C) and Kawasaki disease (KD). BNP: brain natriuretic protein; CAA: coronary artery aneurysm; CD8+: cluster of differentiation 8 (cytotoxic T-cell surface marker); CD177+: cluster of differentiation 177 (neutrophil activation marker); CRP: C-reactive protein; CXCL9, 10, 11: C-X-C motif chemokine ligand 9, 10, 11; GCS: global circumferential strain; GLS: global longitudinal strain; IFN-γ: interferon-gamma; IL-1α: interleukin-1 alpha; IL-1RA: interleukin-1 receptor antagonist; IL-6/10/15/18: interleukin-6/10/15/18; IL-15RA: interleukin-15 receptor alpha; IP-10: interferon-gamma-induced protein 10; LAS: left atrial strain; LMP: luminal myofibroblast proliferation; LV: left ventricle; MAS: macrophage activation syndrome; MCP-1: monocyte chemoattractant protein-1; RANTES: regulated on activation, normal t-cell expressed and secreted; SARS-CoV-2: severe acute respiratory syndrome coronavirus 2; SDF-1: stromal cell-derived factor-1; GM-CSF: granulocyte-macrophage colony-stimulating factor; TRBV11-2 (Vβ21.3): T-cell receptor beta variable 11-2.

Cardiac involvement patterns reflect distinct underlying processes. MIS-C typically presents with widespread myocardial dysfunction requiring vasopressor support, elevated ventricular dysfunction rates, and increased cardiac biomarkers as troponin and brain natriuretic peptide (BNP), indicating a direct cardiomyocyte injury [19]. In the prospective multicenter cohort of Khrongsrattha et al. [9], strain imaging revealed significantly impaired global longitudinal, circumferential, and atrial strain parameters in MIS-C compared to KD, with severe myocardial dysfunction occurring in 25.8% of MIS-C patients *versus* 0% in KD within that cohort, suggesting a substantially different acute myocardial burden. KD cardiac inflammation primarily localizes around coronary vessels rather than diffusely affecting myocardium [10]. This coronary predilection does not exclude myocardial involvement entirely: measurable strain abnormalities occur in KD patients irrespective of coronary artery status, and transient coronary dilation is documented in a meaningful minority of MIS-C patients, underscoring that a distinction between the two conditions reflects predominant rather than exclusive phenotypic expression [9,10,11]. Acute myocardial dysfunction is generally milder in KD. Nevertheless, advanced imaging detects persistent subclinical abnormalities despite normal standard echocardiography, likely reflecting ongoing low-grade inflammation and early fibrotic changes in KD [4,10]. A comparation of the divergent pathophysiological pathways involved in MIS-C and KD is shown in Figure 2.

## 4. Myocardial Strain Beyond Ejection Fraction in MIS-C and KD

The LVEF, though historically central to cardiac functional assessment, reflects volumetric rather than intrinsic myocardial mechanics, a distinction that carries consequence in inflammatory cardiomyopathy. Its limitations are especially apparent in MIS-C and KD, where a subclinical myocardial injury may precede measurable changes in chamber volumes or systolic shortening fractions [7,20]. Myocardial strain addresses this gap directly: by quantifying the percentage change in myofibril length during contraction, it captures intrinsic deformation mechanics rather than inferring function from geometrically derived surrogates. The mechanistic superiority of GLS reflects the anatomic distribution of myocardial vulnerability. Longitudinally oriented subendocardial fibers are disproportionately susceptible to inflammatory and ischemic injuries, owing to their location at the termination of the coronary circulation and their exposure to the highest wall stress during systole. Consequently, GLS deteriorates earlier and more sensitively than circumferential or radial parameters when myocardial damage begins [7]. This hierarchy of dysfunction has quantifiable structural correlates: circumferential shortening contributes approximately 67% of the ejection fraction, against 33% for longitudinal shortening, such that circumferential mechanics can substantially preserve a normal LVEF even when longitudinal function is meaningfully compromised [7,21]. Compensatory subepicardial hypertrophy amplifies this masking effect further, demonstrating that ejection fraction can remain at 50% despite significantly impaired GLS when concentric remodelling coexists [7,20].

### 4.1. Strain Abnormalities as a Distinct Myocardial Phenotype

The acute phase of MIS-C generates a pattern of myocardial deformation that is qualitatively and quantitatively distinct from KD, across multiple parameters in independent cohorts of patients [19,22]. In a prospective multicentre cohort directly comparing the two conditions, MIS-C patients demonstrated significantly worse left ventricular GLS, global circumferential strain (GCS), left atrial strain (LAS), and right ventricular free wall strain (RVFWS), alongside impaired diastolic deformation indices, compared to KD patients, despite broadly similar inflammatory presentation at admission [9,19,23]. Approximately 45% of hospitalized patients exhibited a depressed left ventricular strain, which can be measured by either four-chamber longitudinal strain or mid-ventricular circumferential strain, with the nadir occurring at approximately five days into the illness course [24]. Crucially, strain imaging unmasked injury that conventional assessment did not detect among MIS-C patients with preserved ejection fraction: a substantial proportion demonstrated GLS values below −18%, revealing myocardial dysfunction that would remain clinically invisible without deformation analysis [9,24]. The prognostic implications of these findings have been systematically quantified. Multivariable analysis identified GLS, GCS, peak LAS, and RVFWS as independent predictors of myocardial injury, with odds ratios of 1.45, 1.39, 0.84, and 1.59, respectively [19].

The multicentre MUSIC study, analyzing 349 hospitalized MIS-C cases with a median age of 8.7 years via two-dimensional-speckle tracking echocardiography, provided the most rigorously powered evidence to date in demonstrating that admission strain parameters independently predicted in-hospital adverse cardiovascular events, including vasoactive medication requirement, arrhythmias, cardiac arrest, and need of extracorporeal membrane oxygenation [24]. Each one percentage point deterioration in four chamber longitudinal strain increased the odds of adverse outcome by 9% (adjusted OR 1.09, 95%CI 1.07–1.12), while early diastolic longitudinal strain rate reflecting diastolic reserve demonstrated an even stronger association (adjusted OR 1.68, 95%CI 1.26–2.23) [24].

### 4.2. Beyond the Left Ventricle: Atrial and Right Ventricular Strain as Novel Instruments

Strain assessment in MIS-C has increasingly moved beyond the traditional focus on left ventricular systolic function, exposing a more complex and multi-chambered pattern of myocardial involvement. Indeed, peak LAS was identified as a strong diastolic echocardiographic predictor of myocardial injury in MIS-C [19], reflecting its sensitivity to elevated left ventricular filling pressures and atrioventricular coupling impairment even when systolic parameters remain relatively preserved. Right ventricular involvement adds a further dimension of complexity. The correlation between worse longitudinal RVFWS and higher relative IL-8 expression (ρ = −0.59) suggests that right ventricular dysfunction in MIS-C may reflect a peculiar sensitivity to specific cytokine-driven pathophysiological pathways rather than representing a secondary consequence of left-sided hemodynamic compromise [18]. Prospective six-month follow-up studies reveal differential recovery kinetics between chambers: while left ventricular GLS demonstrates progressive improvement between six weeks and six months post-diagnosis, RVFWS remains statistically unchanged across the same time interval [25]. This chamber-specific divergence in recovery trajectory may indicate distinct vulnerability to inflammatory injury, differing fibrotic propensity, or asymmetric hemodynamic loading. Persistent abnormalities may also reflect the increased sensitivity of the right ventricle to pulmonary vascular afterload during and following acute inflammatory phase. These mechanisms remain incompletely understood and warrant dedicated longitudinal surveillance protocols rather than a uniform echocardiographic follow-up strategy.

### 4.3. Subclinical Myocardial Dysfunction Despite Apparent Recovery

KD presents a mechanistically distinct and temporally different pattern of myocardial involvement. Histologically confirmed myocarditis occurs in 50–70% of cases during the acute phase [26], yet this degree of myocardial involvement is frequently underappreciated given KD’s coronary predominant involvement. Left ventricular function recovers rapidly, often within days after IVIG administration, a trajectory attributable not to absence of genuine myocardial inflammation, but to its reversible edema-driven nature rather than structural cardiomyocyte necrosis. This distinction is important, as both conditions involve myocardial inflammation to varying degrees, and the myocardial-*versus*-coronary framework that distinguishes MIS-C from KD reflects predominant rather than exclusive phenotypic expression. Transient coronary dilation in a meaningful minority of MIS-C patients [9,11] and persistent subclinical strain abnormalities in KD patients without coronary involvement [27] together confirm that both conditions occupy overlapping rather than entirely separate pathophysiological territory. Mid-term follow-up studies in KD patients, including those without CAAs, reveal persistently reduced GLS and GCS in comparison with age-matched healthy controls, despite LVEFs remain within normal limits [27]. The independence of this subclinical dysfunction from the coronary artery status is mechanistically significant: it suggests that myocardial injury in KD extends beyond a mere pericoronary inflammation to affect global myocardial mechanics through pathways that conventional risk stratification, anchored on coronary artery Z-scores, does not capture. The correlation of these strain abnormalities with circulating markers of extracellular matrix turnover, including procollagen type III N-terminal propeptide (PIIINP) and procollagen type I C-terminal propeptide (PICP) [27], also suggests that ongoing fibrotic remodelling, with unresolved inflammation, may underlie persistent deformation abnormalities detected at mid-term follow-up assessments. Advanced myocardial work analysis extends this observation further. Pressure-strain loop derived myocardial work indices reveal reduced global work efficiency in KD patients with coronary dilations even when both GLS and LVEF are within normal ranges [28], identifying a layer of subclinical dysfunction that eludes both conventional and standard strain assessment.

## 5. Coronary Artery Z-Scores-Dilation *Versus* Aneurysm in KD and MIS-C

### 5.1. Coronary Artery Involvement: A Shared Metric with Divergent Phenotypes

The most relevant cardiovascular complication of KD is coronary artery involvement, and the Z-score—a body surface area-adjusted age-independent standardized measurement—has become the central metric around which prognosis, treatment intensity, and follow-up frequency are established and organized [29]. In MIS-C, the same metric has also been applied, but the pattern of coronary involvement captured is markedly different in terms of severity. In KD, coronary artery dilation can begin within the second week of illness and, without IVIG treatment, it may progress to frank aneurysm formation in approximately 25% of cases. With IVIG therapy, the incidence of CAAs has fallen to 4–6%, though the risk is never fully eliminated [14]. The current American Heart Association (AHA) classification, updated in 2024, stratifies KD coronary involvement by maximum Z-score into five categories with directly corresponding management intensities—from routine surveillance at the normal end to lifelong antiplatelet/anticoagulation therapy and structured angiographic follow-up at the giant aneurysm end (see Table 1) [14]. The maximum Z-score reached during the acute illness is, consistently across studies, the strongest predictor of long-term outcome identified to date [10].

### 5.2. Lower Prevalence and Milder Severity of Coronary Artery Involvement in MIS-C

Coronary artery abnormalities occur in MIS-C but with a considerably different epidemiological signature. A systematic review reported that coronary dilations are observed in a minority of MIS-C patients, and that progression to true CAAs and particularly to giant aneurysms is less frequent than in untreated KD [30]. A further cohort study similarly documented that while coronary involvement was present across their MIS-C cohort, but the majority of abnormalities fell within the dilation or small aneurysm range [30,31,32]. This predominance of transient lower-grade Z-scores rather than high-grade structural aneurysm formation appears to be a consistent and distinguishing feature of MIS-C coronary pathophysiology [31].

### 5.3. Natural History and Regression Patterns of Coronary Abnormalities in KD

The natural history of KD-related aneurysms is rather well-characterized. A pooled analysis including data from 21 studies and over 10,000 KD patients confirmed that small aneurysms carry near-universal regression rates and excellent prognosis, while giant aneurysms do not truly regress structurally, carrying a lifelong risk of occlusion, myocardial infarction, and sudden death [29]. Even apparent luminal normalization on imaging does not represent a true vascular recovery, as permanent intimal thickening and abnormal vascular reactivity persist over time [14,29]. In MIS-C, by contrast, most coronary artery abnormalities identified acutely show resolution on the follow-up, as documented across multiple cohorts of patients [33,34,35].

### 5.4. Challenges in Z-Score Standardization and Functional Assessment

A critical but underappreciated challenge complicating accurate comparison across both KD and MIS-C patients is the lack of Z-score formula standardization. A systematic comparison between the Boston, Kobayashi, Dallaire, and Olivieri systems found that up to 22% of patients could receive a different management classification, including anticoagulation decisions, depending solely on which formula was applied [36]. This variability affects KD management directly and likely contributes to inconsistent reported rates of coronary involvement in the medical literature related to MIS-C, where formula choice across centres is rarely harmonized [14,36]. Beyond classification, functional interrogation of coronary lesions matters. Exercise stress echocardiography has been shown to detect inducible ischemia in KD patients with aneurysms whose resting studies appeared reassuring, underscoring the gap between structural and functional recovery [37]. Pharmacological modification of regression trajectory has also been explored: renin-angiotensin-aldosterone system inhibitors may promote aneurysm regression in KD, but this suggestion remains preliminary and is not considered by current guidelines [38]. Whether analogous strategies apply to the smaller subset of MIS-C patients with persistent aneurysms remains entirely unstudied.

## 6. Comparative Outcomes and Longitudinal Recovery in KD and MIS-C

The most direct comparative evidence derives from a multicenter prospective cohort study that examined long-term cardiovascular outcomes in 67 children with MIS-C and 61 with KD at 1–3 years post-diagnosis [9]. Their findings establish a rather clear pattern: KD patients, particularly those with medium or large CAAs, carry a substantially greater burden of persistent cardiovascular abnormalities at a longer-term follow-up, while MIS-C patients demonstrate a complete recovery across most measured parameters, including LVEF and LVGLS [10]. Coronary abnormalities in MIS-C predominantly resolve during follow-up, contrasting sharply with the KD medical literature in which medium and giant aneurysm patients carry permanent coronary sequelae, requiring lifelong surveillance and antithrombotic therapy [29]. In a single-centre cohort study, persistent long-term GLS abnormalities in a subset of MIS-C patients were found to correlate significantly with cardiac troponin levels at the time of acute illness, suggesting that the degree of initial myocardial damage may determine the completeness of long-term functional recovery [39]. This troponin-strain correlation introduces an important stratification principle: those with the highest acute myocardial injury burden may warrant prolonged strain-based surveillance, similar to what is routinely applied in KD. Table 2 compares coronary and myocardial outcomes in KD and MIS-C, respectively.

Taken together, the weight of current evidence suggests MIS-C may have generally more complete cardiovascular recovery than KD, but one where a biologically distinct high-risk subset exists whose long-term trajectory remains incompletely defined. KD, particularly at the medium and giant aneurysm end of the spectrum of KD-related complications, continues to represent the highest long-term cardiovascular risk condition, with subclinical myocardial dysfunction persisting even in patients without aneurysms.

**Figure 3:**
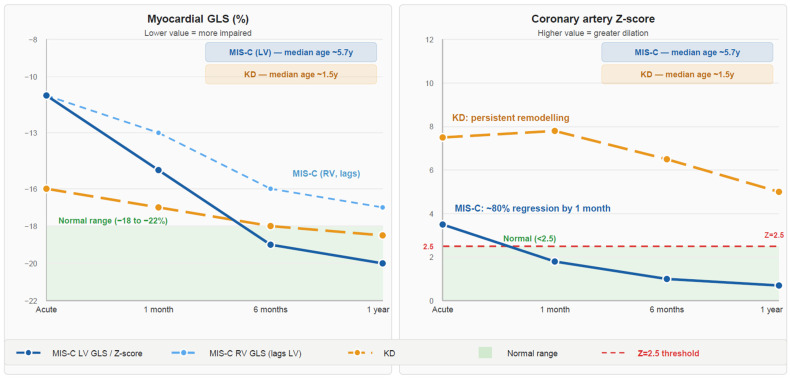
Schematic representation of divergent temporal trajectories of myocardial global longitudinal strain (GLS) and coronary artery Z-scores in multisystem inflammatory syndrome in children (MIS-C) and Kawasaki disease (KD) across the acute phase, 1 month, 6 months, and 1 year of follow-up. Median ages reflect whole-cohort values from the included studies: MIS-C median age 5.7 years (IQR 3.0–9.4); KD median age 1.5 years (IQR 1.1–2.8). Age-stratified strain subgroup data were not available from the included studies; trajectories represent aggregate cohort findings and should be interpreted with awareness of the inherent age-related confound between the two populations. Trajectories are schematic approximations based on representative cohort data and do not represent pooled quantitative estimates. *Left panel:* GLS trajectories showing severe acute impairment with substantial left ventricular (LV) recovery in MIS-C *versus* milder but persistent subclinical impairment in KD; right ventricular (RV) recovery in MIS-C lags behind LV recovery at all post-acute timepoints. *Right panel:* coronary artery Z-score trajectories showing rapid regression to within normal range in MIS-C *versus* persistent elevation in KD with gradual remodelling. GLS: global longitudinal strain; LV: left ventricle; RV: right ventricle.

The recovery patterns and long-term cardiovascular differences outlined above should not obscure the fact that MIS-C and KD share overlapping inflammatory cardi-ovascular mechanisms, including myocardial inflammation and vascular injury. The distinctions described reflect differences in the anatomical predominance, severity, and durability of injury in each condition rather than wholly separate disease processes. The persistent subclinical myocardial dysfunction in KD and the transient coronary involvement in a proportion of MIS-C patients are both expressions of this shared inflammatory substrate, and this conceptual nuance is relevant to how condition-specific surveillance frameworks are interpreted and applied. Figure 3 shows the divergent temporal trajectories of myocardial GLS and coronary artery Z-scores in MIS-C and KD.

## 7. Discussion

The long-term cardiovascular trajectories of MIS-C and KD suggest how two phenotypically convergent post-infectious syndromes can diverge fundamentally in both mechanism and prognosis. What presents at the bedside as a shared constellation of fever, systemic inflammation, and cardiovascular involvement may evolve into two pathologically distinct entities with deviating implications for surveillance and management. The incidence of MIS-C has declined since the emergence of Omicron variants of the SARS-CoV-2 virus, likely due to genetic differences in the virus itself and population immunity to SARS-CoV-2 from both immunization and infection, but MIS-C-like syndromes have been reported in association with other viral infections [40]. The behaviour of the myocardium provides the most clinically instructive contrast with KD. In MIS-C, myocardial dysfunction is a defining feature of the acute illness, frequently severe enough to require hemodynamic support and reflected in disproportionate elevation of troponin and NT-proBNP [9,19]. Rapid identification of patients with MIS-C pathophysiology remains critical to aid the differential diagnosis with macrophage activation syndrome [41]. Yet, acute MIS-C severity does not predict persistence: strain parameters, while markedly abnormal at presentation of MIS-C, demonstrate a substantial recovery in a few months [25,42]. The specific dissociation in recovery kinetics, including RVFWS remaining statistically unchanged across an interval during which left ventricular GLS continues to improve, has not been mechanistically explained and constitutes one of the most important unresolved questions in MIS-C follow-up [25]. Whether this reflects differential cytokine-mediated vulnerability, asymmetric hemodynamic loading during the acute phase, or intrinsic differences in right ventricular fibrotic propensity remains unknown.

On the contrary, KD is a highly variable childhood vasculitis reported worldwide and interlinked with both environmental and genetic factors, in which IVIG resistance and CAA development may vary across different geographic areas and according to the size of patient’s inflammatory response [43]. A unique explanation of its etiopathogenesis is currently unknown, though an abnormal release of pro-inflammatory cytokines giving rise to paramount inflammation makes KD quite comparable to multifactorial autoinflammatory disorders [44,45]. In KD, the acute myocardial insult is comparatively milder and attributable primarily to interstitial edema rather than cardiomyocyte necrosis, a mechanistic distinction that accounts for its characteristically rapid functional recovery following IVIG administration. This apparent normalization may however be misleading, as speckle-tracking echocardiography consistently unmasks persistent subclinical deformation abnormalities in KD irrespective of coronary artery status, and the significant negative correlations between GLS and circulating markers of extracellular matrix turnover, including PIIINP and PICP, which indicate that low-grade myocardial fibrosis continues well beyond the point at which standard indices have normalized [27]. A further interpretive consideration is the hemodynamic effect of acute-phase therapies. Intravenous immunoglobulin, administered as a high-volume infusion, may transiently increase preload and influence strain parameters, particularly left atrial strain, which is sensitive to filling pressures [19,24]. Corticosteroids and other immunomodulatory treatments may further contribute to variability in the hemodynamic status. Since echocardiography is often performed during acute or early recovery phases, these treatment-related effects should be considered potential confounders of strain measurements.

Cardiac magnetic resonance (CMR) imaging, through late gadolinium enhancement and T1/T2 mapping, offers tissue-level characterization inaccessible to strain imaging alone, and may prove the most clinically impactful modality for confirming whether persistent strain deficits in KD and MIS-C reflect truly irreversible myocardial fibrosis [4,29]. Prospective studies integrating serial CMR with speckle-tracking echocardiography are therefore a priority for establishing which patients harbour permanent myocardial injury. The coronary trajectories present an equally instructive contrast, though in the opposite direction. In KD, the three-phase coronary vasculopathy carries implications that extend well beyond acute illness: a stenotic risk persists even in angiographically normalized vessels, and this structural reality appropriately drives the intensity and duration of antiplatelet and anticoagulation strategies [10,13]. In MIS-C, the approximately 80% regression rate of coronary abnormalities reflects a fundamentally less destructive vascular injury, mechanistically attributable to transient immune complex deposition rather than sustained transmural inflammation, which defines KD vasculitis [9,14]. This distinction is not merely descriptive: it carries potential management implications.

In KD, coronary Z-score trajectories appropriately drive treatment intensity and determine the duration of antiplatelet or anticoagulation therapies. In MIS-C, the long-term follow-up may benefit from a shift towards ongoing myocardial strain assessment rather than coronary surveillance: each one-percentage-point deterioration in four-chamber longitudinal strain independently conferred a 9% increase in adverse event odds, while GLS was identified as a strong predictor of myocardial injury [19,24]. In KD, the demonstration of abnormal myocardial work index even in cases with normal ejection fraction suggests this tool as a promising frontier in detecting the earliest signature of subclinical dysfunction [28].

Several important uncertainties constrain the translation of these findings into uniform clinical practice. The optimal duration and frequency of strain-based surveillance in both MIS-C and KD remain undefined by prospective data, and the available evidence is predominantly derived from short- to intermediate-term follow-up intervals of weeks to months, with truly long-term adult outcomes remaining uncharacterized for both conditions. The biological basis for differential recovery in MIS-C and whether persistent RVFWS abnormalities confer meaningful long-term cardiovascular risk require prospective cohort data with follow-up extending beyond the currently available six-month window. In KD, the relationship between subclinical abnormalities detected at mid-term follow-up assessments and adverse cardiovascular events in adulthood remains unanswered, as existing longitudinal datasets rarely extend beyond adolescence. The clinical instinct to manage both the conditions similarly based on their shared inflammatory context and overlapping acute presentation risks may under-detect the persistent myocardial vulnerability that characterizes MIS-C recovery and underappreciates the structural coronary risk that may persist in KD long after clinical resolution. Recognizing these opposite trajectories is not an academic distinction: it is the evidence basis on which syndrome-specific mechanistically informed follow-up protocols should be constructed.

## 8. Limitations

Several methodological limitations constrain the conclusions drawn in this review and require explicit acknowledgment. A systematic age discrepancy between the two populations of patients represents an inherent confound in cross-condition comparisons. KD predominantly affects children under 5 years of age, whereas MIS-C typically presents in older children with a median age of approximately 8–9 years [9]. Immature myocardium differs from that of older children in collagen composition, fibrotic response capacity, and intrinsic deformation mechanics as well as in strain magnitude, recovery kinetics, and remodelling, which may partly reflect age-related differences in myocardial substrate rather than disease-specific biology alone [27]. This limitation highlights the need for age matched normative strain data in future prospective studies.

Speckle-tracking echocardiography carries important methodological constraints that substantially affect cross-study comparability: strain measurements are sensitive to image quality and loading conditions, and tachycardia during the acute febrile phase may independently alter values irrespective of true myocardial dysfunction. Universally validated pediatric normative reference ranges stratified by age, sex, and body surface area remain unavailable, meaning that prognostic thresholds applied across the reviewed studies are largely extrapolated from adult populations or single-centre reference cohorts, a limitation that affects the clinical translatability of absolute strain values in both conditions. Acute therapies further complicate interpretation: IVIG-associated volume loading in KD and immunomodulatory agents in MIS-C may independently affect and thereby influence strain measurements, with left atrial strain being particularly vulnerable to acute hemodynamic shifts [19].

The narrative design of this review, while appropriate for synthesizing a heterogeneous medical literature, does not permit formal quality appraisal, bias assessment, or quantitative pooling of effect estimates. The included studies vary substantially in design, sample size, imaging methodology, and follow-up duration, and collective interpretation of their findings carries an inherent risk of overestimating consistency across cohorts.

A further limitation concerns the evolving definition of myocardial involvement itself. The 2025 guidelines of the European Society of Cardiology introduce inflammatory myopericardial syndrome (IMPS) as an ‘umbrella’ term spanning the spectrum from isolated myocarditis to isolated pericarditis, including overlap forms, to be applied during initial diagnostic evaluations. Since the studies included here predate or were conducted independently of this framework, myocardial involvement was defined heterogeneously across cohorts. Consequently, reclassification under this novel IMPS paradigm may affect the comparability of strain and coronary Z-score findings across studies.

Finally, the available evidence is predominantly derived from short- to intermediate-term follow-up intervals of weeks to months, with few studies extending beyond mid-term assessment. Therefore, conclusions regarding persistent myocardial dysfunction in MIS-C and subclinical coronary remodelling in KD are necessarily constrained to these time horizons. Whether subclinical strain abnormalities in either condition translate into clinically significant cardiovascular events in adulthood remains unanswered and requires prospective registry-based cohort studies with follow-up extending well beyond adolescence.

## 9. Conclusions

MIS-C and KD share a post-infectious hyperinflammatory phenotype, but have a tendency to follow divergent long-term cardiovascular trajectories, supporting condition-specific surveillance strategies. KD imposes its greatest burden through structural coronary injury, a process that persists histologically long after clinical resolution and standard imaging appears reassuring. MIS-C appears predominantly characterized by myocardial involvement, with coronary abnormalities being largely transient. Yet a clinically important subgroup retains depressed GLS after ejection fraction has normalized, with acute troponin elevation which may help identify those at higher risk of incomplete myocardial recovery. In both conditions, conventional echocardiographic assessment systematically underestimates any residual injury. These contrasting trajectories support reorienting MIS-C follow-up towards prolonged strain-based surveillance in high-risk patients, while KD management continues to prioritize structured coronary imaging stratified by AHA Z-score classification.

Translating these findings into clinical practice will require addressing three interconnected research priorities. First, the lack of a universally adopted Z-score formula introduces classification inconsistency that directly alters risk stratification and treatment thresholds; prospective validation of a standardized formula across ethnically diverse pediatric populations represents an achievable and high-impact target. Second, pediatric-specific strain reference ranges stratified by age, sex, and body surface area would be necessary to define meaningful prognostic thresholds, and their development through large normative cohort studies would transform strain from a research instrument into a reliably actionable decision tool. Third, the field currently lacks prospective longitudinal data extending beyond six months in MIS-C and beyond adolescence in KD, precisely the time horizons at which subclinical myocardial fibrosis and coronary remodelling may produce their first clinically detectable consequences. Multicentre registry-based cohort studies incorporating serial strain imaging, coronary artery Z-score trajectories, and cardiac biomarker profiling may offer the most reliable pathway to resolve the outstanding questions this analysis has identified.

## Figures and Tables

**Table 1:** American Heart Association classification of coronary artery involvement by maximum Z-score in Kawasaki disease.

Category	Z-Score Threshold	Absolute Diameter	Management Implication
No involvement	<2	—	Routine follow-up; no antiplatelet therapy required
Dilation	2 to <2.5	—	Low-dose aspirin; short-term echocardiographic follow-up
Small aneurysm	≥2.5 to <5	—	Low-dose aspirin; extended follow-up with echocardiography
Medium aneurysm	≥5 to <10	<8 mm	Dual antiplatelet therapy or anticoagulation; regular long-term follow-up
Large/giant aneurysm	≥10	≥8 mm	Anticoagulation; lifelong surveillance; risk of thrombosis, stenosis, myocardial ischemia and sudden death

**Table 2:** Comparative coronary and myocardial outcomes in Kawasaki disease *versus* multisystem inflammatory syndrome in children: the surveillance recommendations presented in this table represent a synthesis of current expert opinions and emerging evidence; they are intended as a framework to guide clinical decision-making and do not constitute formally evidence-established guidelines.

*Parameter*	KD	MIS-C
Coronary involvement rate (untreated)	25% develop aneurysms without IVIG; 4–6% with IVIG	Predominantly dilation-range Z-score; true aneurysm uncommon
Typical peak Z-score range	Variable; giant aneurysms (Z ≥ 10) well-documented	Mostly dilation (Z 2.5–5); rarely medium or large aneurysm
Giant aneurysm risk	Significant; permanent structural vessel damage	Rare; not a defining feature of MIS-C coronary phenotype
Coronary regression on follow-up	Small/medium: high regression rates; giant: structural changes persist lifelong	Most dilation resolves; favourable trajectory in majority of cohorts
Acute GLS depression	Moderate; inflammation- and ischemia-mediated	Greater than KD; predominantly diffuse myocarditis pattern
Myocardial strain recovery	Subendocardial strain remains reduced in chronic phase even without aneurysms	Generally favourable; subset with persistent GLS abnormality correlates with troponin T
Long-term cardiovascular risk	Higher; especially with medium/giant aneurysms; lifelong surveillance required	Lower overall; but incompletely defined in high-troponin subgroup
Suggested surveillance	Echocardiography + stress testing; coronary angiography for giant aneurysms	Echocardiography with GLS; prolonged strain-based follow-up for high-troponin patients

GLS: global longitudinal strain; IVIG: intravenous immunoglobulin; KD: Kawasaki disease; MIS-C: multisystem inflammatory syndrome in children.

## Data Availability

No new data were created or analyzed in this study.

## Abbreviations

The following abbreviations have been used in this manuscript:

AHA	American Heart Association
BNP	brain natriuretic peptide
CAA	coronary artery aneurysm
CCL5 or RANTES	chemokine C-C motif ligand 5
CMR	cardiac magnetic resonance
COVID-19	coronavirus disease 2019
GCS	global circumferential strain
GLS	global longitudinal strain
GM-CSF	granulocyte-macrophage colony-stimulating factor
IFN	interferon
IL	interleukin
IL-1RA	interleukin-1 receptor antagonist
IL-15RA	interleukin-15 receptor antagonist
IMPS	inflammatory myopericardial syndrome
IP-10 or CXCL10	IFN-induced protein 10
IVIG	intravenous immunoglobulin
KD	Kawasaki disease
LAS	left atrial strain
LMP	luminal myofibroblastic proliferation
LVEF	left ventricular ejection fraction
NT-proBNP	N-terminal pro-B-type natriuretic peptide
MCP-1	monocyte chemoattractant protein-1
MIS-C	multisystem inflammatory syndrome in children
PICP	procollagen type I C-terminal propeptide
PIIINP	procollagen type III N-terminal propeptide
RVFWS	right ventricular free wall strain
SARS-CoV-2	severe acute respiratory syndrome coronavirus 2
SDF-1 or CXCL12	stromal cell-derived factor 1
